# Stromal hyaluronan accumulation is associated with low immune response and poor prognosis in pancreatic cancer

**DOI:** 10.1038/s41598-021-91796-x

**Published:** 2021-06-09

**Authors:** Kyösti Tahkola, Maarit Ahtiainen, Jukka-Pekka Mecklin, Ilmo Kellokumpu, Johanna Laukkarinen, Markku Tammi, Raija Tammi, Juha P. Väyrynen, Jan Böhm

**Affiliations:** 1 Department of Surgery, Central Finland Health Care District, Jyväskylä, Finland; 2 Faculty of Medicine and Health Technology, Tampere University, Tampere, Finland; 3 Department of Pathology, Central Finland Health Care District, Jyväskylä, Finland; 4 Department of Education and Research, Central Finland Health Care District, Jyväskylä, Finland; 5 Sport&Health Sciences, University of Jyväskylä, Jyväskylä, Finland; 6 Department of Gastroenterology and Alimentary Tract Surgery, Tampere University Hospital, Tampere, Finland; 7 Institute of Biomedicine, School of Medicine, University of Eastern Finland, Kuopio, Finland; 8 Cancer and Translational Medicine Research Unit, Medical Research Center Oulu, University of Oulu and Oulu University Hospital, Oulu, Finland

**Keywords:** Cancer, Immunology, Gastroenterology, Oncology

## Abstract

Hyaluronan (HA) accumulation has been associated with poor survival in various cancers, but the mechanisms for this phenomenon are still unclear. The aim of this study was to investigate the prognostic significance of stromal HA accumulation and its association with host immune response in pancreatic ductal adenocarcinoma (PDAC). The study material consisted of 101 radically treated patients for PDAC from a single geographical area. HA staining was evaluated using a HA-specific probe, and the patterns of CD3, CD8, CD73 and PD-L1 expression were evaluated using immunohistochemistry. HA staining intensity of tumour stromal areas was assessed digitally using QuPath. CD3- and CD8-based immune cell score (ICS) was determined. High-level stromal HA expression was significantly associated with poor disease-specific survival (*p* = 0.037) and overall survival (*p* = 0.013) In multivariate analysis, high-level stromal HA expression was an independent negative prognostic factor together with histopathological grade, TNM stage, CD73 positivity in tumour cells and low ICS. Moreover, high-level stromal HA expression was associated with low ICS (*p* = 0.017). In conclusion, stromal HA accumulation is associated with poor survival and low immune response in PDAC.

## Introduction

Pancreatic ductal adenocarcinoma (PDAC) is one of the most aggressive solid malignancies with 5-year survival rates of 2–9%^1,2^. This is partly related to advanced disease stage at the time of diagnosis ruling out curative surgery. Tumour cells are in constant interaction with non-neoplastic cells, and the tumour microenvironment influences cancer progression. PDAC has been shown to develop mechanisms that suppress the host immune response against the tumour^3^. The first signs of this immune suppression are seen already in the premalignant lesions^4^. PDAC is characterized by an abundant desmoplastic stroma, which has been suggested to facilitate the escape from the immune surveillance^5,6^.

Hyaluronan (HA) is one of the main components of the extracellular matrix. In normal physiological conditions, it is strongly expressed during wound healing and at sites of inflammation, including cancer^7^. It also influences immune responses^8^. A complex regulation system controls HA metabolism, mainly dependent on HA-producing synthases and degrading hyaluronidases. The activation of the cell surface HA receptors such as CD44^9^ and RHAMM^10^ modulate cell proliferation, aggregation, migration and angiogenesis^11^ and may also be involved in the HA-induced epithelial–mesenchymal transition^12^ and stem cell functions^13^. HA has been shown to be overexpressed in most human malignancies^14–23^. Several studies have indicated that hyaluronan accumulation in the tumour cells and/or peritumoral stroma is related to tumour progression and poor survival in many cancer types^23–31^. However, the mechanisms underlying the association between accumulation of HA, host immune response and poor survival remain unclear, especially in PDAC.

The association between a strong immune response and better survival is well established in various cancers^32–36^. We have previously introduced a T-lymphocyte-based immune cell score (ICS) as a strong favourable prognostic factor in PDAC^37^. Extensive alterations occur in the complex PDAC microenvironment during the tumorigenesis. Multiple mechanisms, such as overexpression of the immunosuppressive molecules CD73 and PD-L1, may lead to immune suppression^38–41^. There is some evidence showing that HA plays a role in immune response regulation^42,43^. According to our hypothesis this might be one of the key factors explaining the association between HA accumulation and low survival among cancer patients^25^.

The aim of the present study was to examine the prognostic role of stromal HA accumulation and its relation to immune cell infiltration and the immune-suppressing molecules CD73 and PD-L1 in PDAC.

## Methods

### Patients

From 2000 to 2016, a total of 129 patients with PDAC were operated on with curative intent in the Central Hospital of Central Finland, Jyväskylä, Finland. Patients with locally inoperable tumour, peritoneal carcinosis or distant metastases were excluded, resulting in the 101 patients with stage IA-IIB disease. Detailed information on patient and tumour characteristics, surgical treatment and complications, oncological treatment and follow-up were collected prospectively, updated and confirmed by a review of patient records review. None of the included patients received neoadjuvant chemotherapy before surgery.

### Histopathological examination

All histopathological tumour specimens were reviewed by an experienced gastrointestinal histopathologist (JB). Tumour staging was done according to the 7th edition of the UICC/AJCC TNM categories^44^. The grading was performed according to the WHO classification of tumours 2010^45^.

### Tumour sampling, HA assay, and immunohistochemistry

Tissue microarray (TMA) blocks were constructed as described previously, from formalin-fixed paraffin-embedded primary PDAC patient tumour samples. Two tissue cores 0.6 mm in diameter were taken both from the core of the tumour and the invasive margin from representative tumour blocks. Sections of 2 µm thickness were used for immunohistochemical (IHC) analyses^37^.

**Figure 1:**
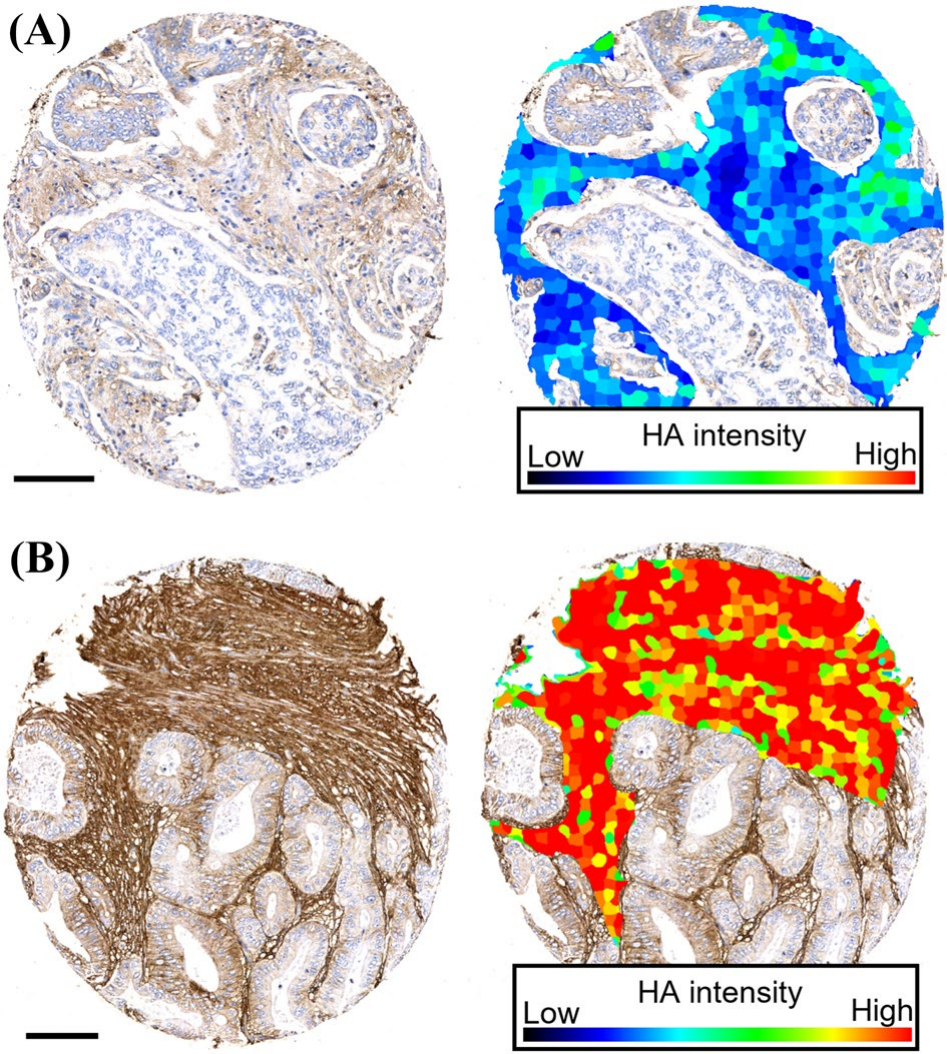
Representative examples of tissue microarray cores with low (**A**) and high (**B**) stromal hyaluronan (HA) intensity. The images show the exclusion of the tumour parenchyma and the delineation of the superpixels formed by the program used for analysis. Scale bars indicate 100 µm.

**Figure 2:**
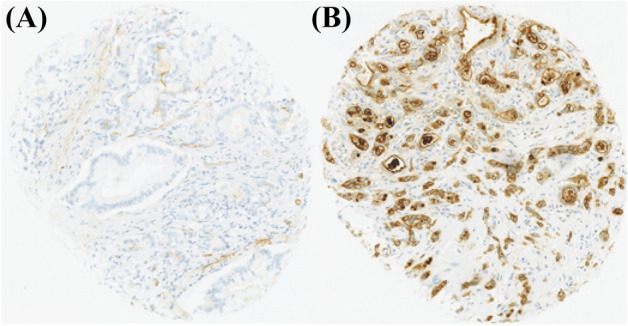
Representative examples of tissue microarray cores with low (**A**) and high (**B**) expression of CD73.

**Figure 3:**
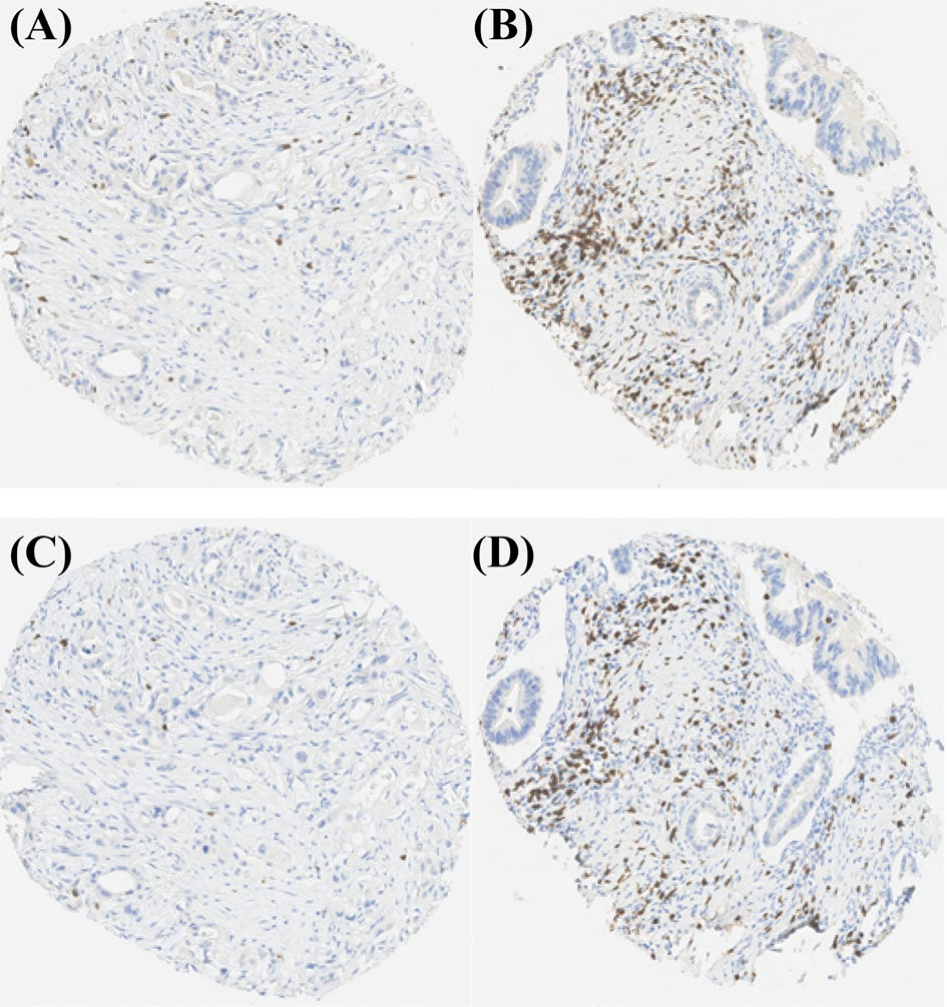
Representative examples of tissue microarray cores with low (**A**) and high CD3+ (**B**), and CD8+ (**C**,**D**) T-cell densities.

**Figure 4:**
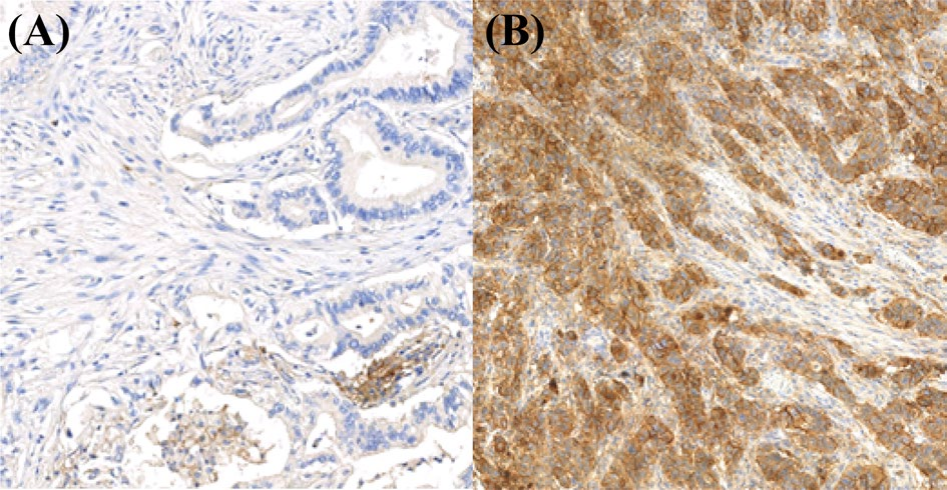
Representative examples of PD-L1 negative (**A**) and positive (**B**) tumour samples.

Hyaluronan was stained as described previously^26^. Briefly, a complex containing the G1 domain of cartilage aggrecan and link protein was labeled with biotin (bHABC), diluted to 3 µg/ml of 1% bovine serum albumin in phosphate buffer, and incubated overnight at 4 °C on sections pretreated with H_2_O_2_ and 1% bovine serum albumin to block endogenous peroxidases and unspecific binding, respectively. After one hour incubation in avidin–biotin–peroxidase (Vector Laboratories, Irvine, CA; 1:200 dilution) the sections were washed with PBS, and incubated in 0.05% 3,3′-diaminobenzidine (Sigma Chemical Co., St. Louis, MO) and 0.03% H_2_O_2_ in the phosphate buffer, followed by nuclear counterstaining with Mayers hematoxylin (Fig. 1). Staining for CD73 was conducted as described previously, with rabbit monoclonal anti-CD73 antibody (D7F9A, Cell Signalling) and ultraView Universal DAB detection kit (Roche) for Ventana^39^ (Fig. 2). Staining for CD3 and CD8 was conducted with anti-CD3 (LN 10, 1:200; Novocastra) and anti-CD8 (SP16, 1:400; Thermo Scientific) antibodies, using a Lab Vision Autostainer 480 (Immunovision Technologies Inc.) (Fig. 3). Staining for PD-L1 was conducted as described previously, with anti-PD-L1 (E1L3N, 1:100; Cell Signalling Technology) antibody, using a BOND-III stainer (Leica Biosystems). PD-L1 staining was carried out using whole tissue sections ^37^ (Fig. 4).

Signal visualization for all IHC was done by diaminobenzidine and sections were counterstained with haematoxylin.

In general, HA staining was clearly seen in all specimen both in stroma and in tumour epithelium. Stained TMA sections were scanned using an Aperio digital slide scanner (Leica Biosystems), followed by analysis using QuPath v 0.1.2 as described below.

ICS was determined using the TMA technique as described earlier^37^. Briefly, ICS describes the immune response represented by the density of CD3 and CD8 positive immune cells in the tumour centre and at the invasive margin. PD-L1 expression was evaluated by estimating the proportion of PD-L1 positivity on the tumour cell surface as described earlier^39^. In addition, we analyzed also the proportion of PD-L1 expression in stromal cells using the 5% staining proportion as a cutoff.

### Quantitative evaluation of HA staining

HA was evaluated using QuPath v 0.1.2^46^. First, the stain vectors and background values were estimated using the *Estimate stain vectors* command to facilitate stain separation with the color deconvolution method. *Simple tissue detection* command was used to delineate the tissue area from the white background. This area was manually edited with the brush tool to exclude tumour epithelial regions. *SLIC superpixel segmentation* was used to divide the area into superpixels (neighboring groups of pixels sharing similar characteristics). DAB intensity was calculated for each superpixel, and the data were exported at individual superpixel level. R statistical programming language version 3.5.2 (R Foundation for Statistical Computing, Vienna, Austria) was used to summarize the mean intensity for each case. The distribution of DAB intensities was similar in different TMAs suggesting that the assay had performed uniformly (Fig. S.1). The cores from tumour centre and invasive margin had a strong positive correlation (Spearman’s rho = 0.69), and average HA intensity of all available tumour regions was therefore used in the main analyses.

Samples were divided into two groups based on the mean intensity value: high and low stromal HA expression. To determine cut-off values for HA expression with optimal sensitivity and specificity, we used receiver operating characteristic (ROC) curve drawn in relation to disease-specific 3-year mortality.

### Statistical analyses

**Figure 5:**
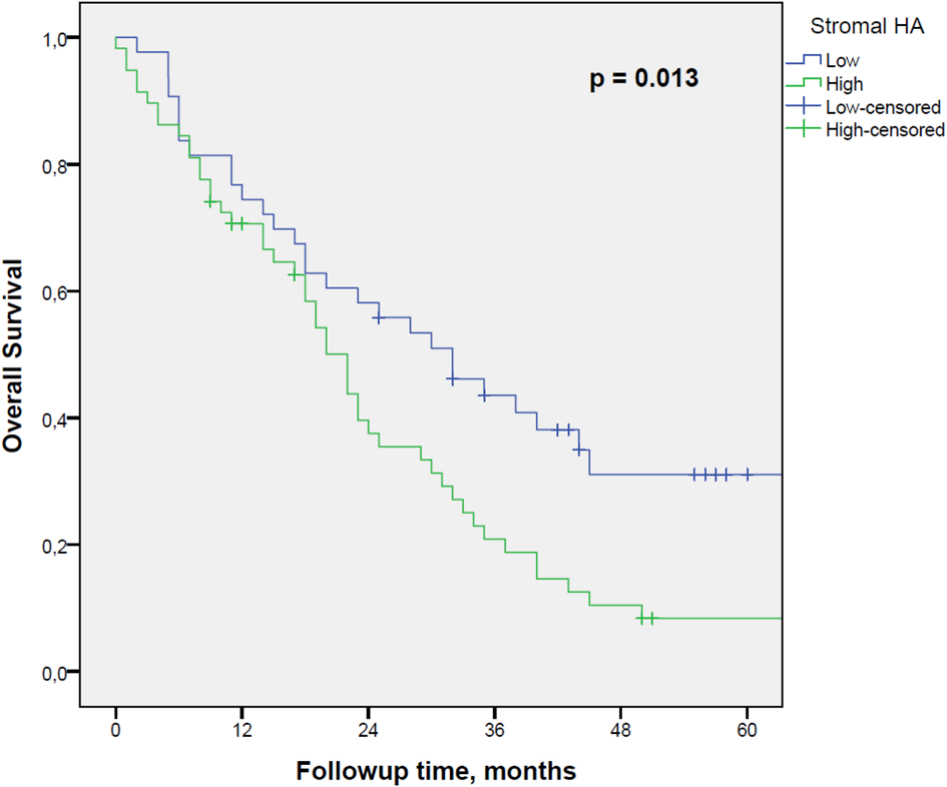
Prognostic impact of stromal HA content on survival on OS.

The chi-square test was used when analysing the associations between HA and clinical and histopathological variables, CD73 positivity and PD-L1 positivity in tumour cells. The estimates for hazard ratios for overall survival (OS) and disease specific survival (DSS) were calculated using univariate and multivariate Cox proportional hazards regression model. Only variables with *p* < 0.05 in univariate analysis were entered into the multivariate analysis despite the a-priori determined confounder, tumour stage (*p* = 0.117). All statistical tests were two-sided. A *p* value less than 0.05 was considered significant. The statistical analyses were performed and Fig. 5 created with IBM SPSS statistics 24 for Windows (IBM Corporation, Armonk, NY, USA, https://www.ibm.com/analytics/spss-statistics-software).

### Compliance with ethical standards

The use of patient samples and the data inquiry were approved by the Oulu University Hospital Ethics Committee. The need to obtain written or oral consent from patients to use their samples in research was waived by the Finnish National Authority for Medicolegal Affairs (VALVIRA, Dnro 10,832/06.01.03.01/2014). This study was designed and performed according to the reporting recommendations for tumour marker prognostic studies (REMARK) and the Declaration of Helsinki^47,48^.

## Results

### Patient demographics

A total of 101 of PDAC patients were included in this study. The distribution of key clinicopathological variables among these patients is shown in Table 1.

**Table 1:** Clinicopathological characteristics.

Total, n	101
**Age, years**	
Median	67
Range	45–82
**Gender, n (%)**	
Male	53 (52.5)
Female	48 (47.5)
**T-stage, n (%)**	
pT1	3 (3.0)
pT2	22 (21.8)
pT3	76 (75.2)
pT4	0 (0)
**N-stage, n (%)**	
pN0	30 (29.7)
pN1	71 (70.3)
**Stage, n (%)**	
IA	3 (3.0)
IB	7 (6.9)
IIA	20 (19.8)
IIB	71 (70.3)
**Histological grade, n (%)**	
1	29 (28.7)
2	60 (59.4)
3	7 (6.9)
Unknown	5 (5.0)
**Perineural invasion, n (%)**	
Positive	33 (32.7)
Negative	64 (63.4)
Unknown	4 (4.0)
**PD-L1 in tumour cells, n (%)**	
Positive	3 (3.0)
Negative	98 (97.0)
**PD-L1 in tumour stroma, n (%)**	
Positive	12 (11.9)
Negative	89 (88.1)
**Immune cell score, n (%)**	
Low	65 (64.4)
High	34 (35.6)
**Stromal hyaluronan expression, n (%)**	
Low	43 (42.6)
High	58 (57.4)
**CD73 expression in tumour cells, n (%)**	
Low	67 (66.3)
High	34 (33.7)

### Associations between stromal HA expression and other histopathological variables

Stromal HA accumulation appeared to associate with low ICS (*p* = 0.017). The associations between stromal HA expression and other clinical and histopathological variables, cell-specific CD73 positivity and PD-L1 positivity in tumour cells were also assessed and are shown in Table 2. Stromal HA accumulation was not associated with other clinicopathological parameters, including CD73 positivity in tumour cells and PD-L1 positivity in tumour cells and stromal cells.

**Table 2:** Clinicopathological variables and their association with stromal hyaluronan (HA).

	HA high, n (%)	HA low, n (%)	*p* Value
**Gender**			
Male	33 (56.9)	20 (46.5)	0.301
Female	25 (43.1)	23 (53.5)	
**T-stage**			
pT1	2 (3.4)	1 (2.3)	0.742
pT2	14 (24.1)	8 (18.6)	
pT3	42 (72.4)	34 (79.1)	
pT4	0	0	
**N-stage**			
pN0	19 (32.8)	11 (25.6)	0.435
pN1	39 (67.2)	32 (74.4)	
**Stage**			
IA	2 (3.4)	1 (2.3)	0.979
IB	4 (6.9)	3 (7.0)	
IIA	12 (20.7)	8 (18.6)	
IIB	40 (69.0)	21 (72.1)	
**Histological grade**			
1	16 (30.2)	13 (30.2)	0.994
2	33 (62.3)	27 (62.8)	
3	4 (7.5)	3 (7.0)	
**Perineural invasion**			
Positive	35 (64.8)	29 (67.4)	0.786
Negative	19 (35.2)	14 (32.6)	
**PD-L1 in tumour cells**			
Positive	1 (1.7)	2 (4.7)	0.392
Negative	57 (98.3)	41 (95.3)	
**PD-L1 in tumour stroma**			
Positive	6 (10.3)	6 (14.0)	0.579
Negative	52 (89.7)	37 (86.0)	
**Immune cell score**			
Low	43 (74.1)	22 (51.2)	*0.017*
High	15 (25.9)	21 (48.8)	
**CD73 in tumour cells**			
Low	39 (67.2)	28 (65.1)	0.823
High	19 (32.8)	15 (34.9)	

### Stromal HA accumulation and survival

Regarding the whole study group, the median follow-up time was 44 (IQR 15.0 to 57.0) months for those alive at the end of follow-up. The estimated median OS for all patients was 25 months [95% CI: (17.7–32.3)]. Stromal HA accumulation was significantly associated with poor DSS (*p* = 0.037) and OS (*p* = 0.013) (Fig. 5).

In the multivariate analysis, stromal HA accumulation was an independent negative prognostic factor together with histopathological grade, TNM stage, CD73 positivity in tumour cells and low ICS (Table 3).

**Table 3:** Multivariate analysis with Cox proportional hazard model.

	Univariate analysis (OS)^a^ HR (95% CI)	*p*	Univariate analysis (DSS)^b^ HR (95% CI)	*p*	Multivariate analysis (OS) HR (95%CI)	*p*	Multivariate analysis (DSS) HR (95% CI)	*p*
**Stromal HA**								
Low	1	**0.015**	1	**0.042**	1	**0.019**	1	**0.048**
High	1.80 (1.22–2.88)		1.67 (1.02–2.72)		1.85 (1.11–3.10)		1.71 (1.00–2.92)	
**CD73 (TC)**^c^								
Negative	1	**0.022**	1	**0.029**	1	**0.015**	1	**0.023**
Positive	1.76 (1.09–2.84)		1.76 (1.06–2.93)		2.01 (1.14–3.53)		2.00 (1.10–3.62)	
**Tumor grade**								
1	1	**0.006**	1	**0.013**	1	**0.012**	1	**0.019**
2	1.95 (1.14–3.34)		1.95 (1.12–3.39)		1.99 (1.15–3.44)		2.01 (1.14–3.56)	
3	3.88 (1.54–9.79)		3.69 (1.36–10.01)		3.96 (1.36–11.52)		3.80 (1.18–12.01)	
**TNM stage**								
IA + IB	0.64 (0.28–1.48)	0.234	0.69 (0.30–1.61)	0.117	0.53 (0.21–1.36)	**0.055**	0.58 (0.22–1.52)	**0.036**
IIA	0.55 (0.29–1.06)		0.49 (0.24–0.99)		0.49 (0.25–0.95)		0.41 (0.20–0.85)	
IIB	1		1		1		1	
**ICS**^d^								
High	1	**0.007**	1	**0.008**	1	**0.006**	1	**0.002**
Low	1.99 (1.21–3.30)		2.05 (1.21–3.47)		2.23 (1.26–3.95)		2.54 (1.40–4.63)	

^a^Overall survival.

^b^Disease specific survival.

^c^Tumour cell.

^d^Immune cell score.

## Discussion

In the present study, using a larger, consecutive patient series from a single geographical area of Northern Finland without apparent selection bias, we showed the role of stromal HA accumulation as an independent prognostic factor for poor survival in pancreatic cancer. We also found an association between the HA accumulation and low immune response as judged by the tumour-infiltrating T-cell densities.

While the number of patients in the present work was higher than in previous studies on HA in PDAC^6,24^, an even larger material would probably have allowed a connection between T-cell score and hyaluronan stronger than that now established. The small tissue cores turned out to be quite acceptable for the analysis since in preliminary tests the HA intensities were strongly correlated between different cores of the same tumour even between the tumour centre and invasive margin. The computer-assisted evaluation of HA staining adopted for the present work was felt easier than manual scoring of sometimes minor differences in intensity. It can also be recommended for future studies due to its independence of personal variation between evaluators.

Stromal HA accumulation in malignancies originating from non-stratified epithelium is associated with a poor survival in a number of solid tumours^7^. Indeed, given the large desmoplastic stroma in PDAC, a major role of HA was expected in the progression of this cancer. The idea was further supported for example by a fact that a drug specifically reducing HA synthesis inhibits human PDAC cell growth in vitro and in mice in vivo^49^. However, clinical trials combining enzymatic removal of HA and cytostatic drugs have been disappointing^50,51^ suggesting that it is not just the content of HA that enhances malignant growth. Rather, activated synthesis and concurrent degradation of HA probably provide an environment supporting cancer spreading^7^. This becomes understandable by considering the two opposite influences of HA on cell migration. By its swelling pressure HA gel creates free space for cells to move in, while at the same time blocks attachment to adjacent cells and matrix proteins.

Indeed, the cell surface hyaluronidase TMEM2 is an independent negative prognostic factor in PDAC^52^, demonstrating the importance of HA degradation in PDAC progression. TMEM2 associates to integrins and clears HA to facilitate cancer cell adhesion and migration^53^. Besides facilitating migration in HA-rich matrix the fragments created by hyaluronidase act as a signal that amplify inflammation.

Increasing numbers of studies have shown the impact of HA on the host immune response: It is suggested to protect tumour cells against immune attack by forming peri-cellular coats^54^. Moreover, HA accumulation seems to facilitate tumour-associated macrophage infiltration and their differentiation into the pro-tumoral M2 phenotype^25,42^ with an immunosuppressive effect preventing antitumour immunity by T-cells. Interestingly, in the present study we demonstrate an inverse correlation between T-cell-based ICS and stromal HA accumulation in PDAC. This supports the idea that an HA-rich extracellular matrix not only acts as a shield between T-cells and tumour cells, but also prevents T-cell infiltration in the whole tumour microenvironment. We have previously published a paper showing that CD73 positivity in PDAC cells is a prognostic factor in PDAC independently of ICS and hypothesized that CD73 suppresses immune response by impacting on the activity of the tumour infiltrating lymphocytes rather than their number^39^. This might also explain why stromal HA expression associates with ICS but not with CD73 or PD-L1.

Recently, different phenotypes of ECM-producing cancer-associated fibroblasts (CAFs) have been described, including inflammatory CAFs and myofibroblastic CAFs^55^. Inflammatory CAFs are supposed to be tumour-promoting via immune suppression^56^. In future, it would be reasonable to find out if the HA accumulation associates with the polarization of CAFs, since this would further give some insight into the potential mechanism behind the association between HA and immune status. One possible link between CAF polarisation and HA synthesis is the STAT3-signaling pathway, since the inhibition of STAT3—pathway has been shown to downregulate HA—synthesis^57^ and, on the other hand, to promote differentiation of CAFs into myCAFs^56^.

As far as we know, the association of HA on T-cell immune response has not been studied earlier in PDAC but the present finding clearly warrants further expansion of the studies to obtain a more detailed view of the interactions between HA and lymphocytes in this disease with such a bleak prognosis. In future studies, information of physical properties (for example molecular mass) of HA molecules is also needed, since there are data indicating that molecular weight affects the biological functions of HA molecules^58^.

In conclusion, our study indicates that stromal HA accumulation may be associated with low T cell densities in the PDAC microenvironment, but still represents an adverse prognostic parameter independent of T cell densities, tumour stage, tumour grade, and CD73 expression. The results warrant further definition of the interactions between T-cell immunity and hyaluronan in the tumour microenvironment.

## Supplementary Information

Supplementary Figure.

## Abbreviations

HA: Hyaluronan
ICS: Immune cell score
PDAC: Pancreatic ductal adenocarcinoma
DSS: Disease-specific survival
OS: Overall survival
TMA: Tissue microarray
