# Supplementary figures and images for: Stromal hyaluronan accumulation is associated with low immune response and poor prognosis in pancreatic cancer
Source: Sci Rep. 2021 Jun 9;11:12216. doi: 10.1038/s41598-021-91796-x (PMC8190291; doi:10.1038/s41598-021-91796-x)

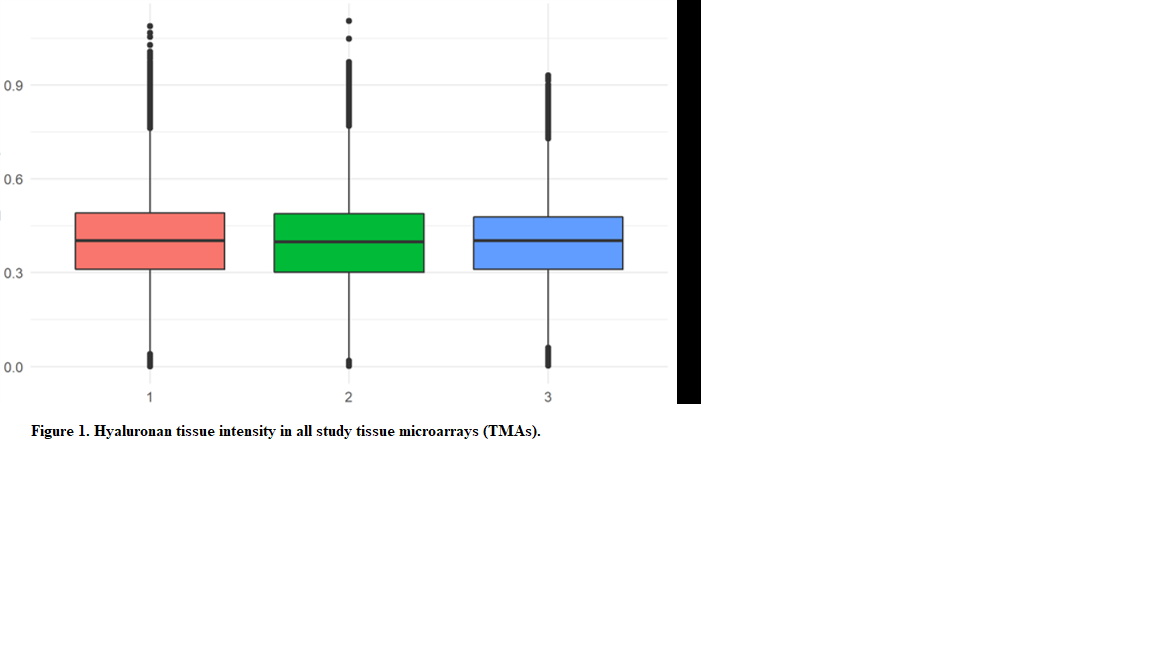

Supplement: Supplementary file 1 — Supplementary Figure. [file 41598_2021_91796_MOESM1_ESM.png]
